# Champion-Led Deprescribing for Persons with Dementia in Primary Care: A Qualitative Study in Accountable Care Organizations

**DOI:** 10.1007/s11606-026-10234-8

**Published:** 2026-02-02

**Authors:** Masami Tabata-Kelly, Lorella G. Palazzo, Jennifer Perloff, Linda Kiel, Michael Parchman, Robert B. Penfold

**Affiliations:** 1 The Heller School for Social Policy and Management, Brandeis University, Waltham, MA USA; 2 Kaiser Permanente Washington Health Research Institute, Seattle, WA USA; 3 Community Health Worker Coalition for Migrants & Refugees, Seattle, WA USA; 4 Center for Surgery and Public Health, Mass General Brigham, Somerville, MA USA

**Keywords:** dementia, accountable care organization, deprescribing, de-implementation, clinical champions

## Abstract

**Background:**

Clinical champions are a known strategy for implementing evidence-based practices; however, their application in de-implementing potentially inappropriate medications (PIMs) among persons with dementia is underexplored. We conducted a pragmatic cluster-randomized clinical trial of a champion-led deprescribing intervention in accountable care organization (ACO) primary care settings.

**Objective:**

To (1) understand clinical champions’ perspectives of their deprescribing projects and (2) identify key contextual factors that influenced champions’ deprescribing PIMs for persons with dementia within ACO primary care.

**Design:**

A qualitative study guided by the de-implementation outcomes framework.

**Participants:**

Clinician champions who engaged in deprescribing projects.

**Approach:**

Data sources included transcripts from monthly learning calls and semi-structured interviews. All champions were invited to participate in learning calls and semi-structured interviews. The 30- to 60-min interviews were conducted using a semi-structured guide. We coded transcripts and performed thematic analysis to identify overarching themes.

**Key Results:**

Eleven champions participated. Feasibility and fidelity of deprescribing were commonly undermined by external and organizational disruptions. Five contextual factors influenced champions’ experiences: limited organizational readiness, lack of information technology infrastructure to support data access and patient identification, the importance of relationship-building and care coordination, the dyadic nature of deprescribing involving care partners, and the pharmacist’s role as a multidisciplinary liaison. Champions employed adaptive, communication- and relationship-centered strategies to support deprescribing efforts.

**Conclusions:**

Champion-led deprescribing for persons with dementia is shaped by key contextual factors within ACO primary care settings. Dementia-specific training helped clinicians tailor deprescribing to local needs, but sustained efforts require supportive organizational structures, including ongoing education, accessible clinical data, and multidisciplinary collaboration. Primary care clinicians are uniquely positioned to lead personalized deprescribing conversations while pharmacists serve as liaisons with providers, patients, and care partners to coordinate deprescribing. Interventions that align with value-based care principles may strengthen system-level coordination and promote safer medication management in dementia care.

**Trial Registration:**

ClinicalTrials.gov ID: NCT05359679

**Supplementary Information:**

The online version contains supplementary material available at 10.1007/s11606-026-10234-8.

## INTRODUCTION

Over five million older adults aged 65 and older in the USA are living with dementia.^1^ As the aging population continues to grow, this number is projected to rise to approximately 14 million by 2060.^2^ Among persons with dementia, the use of potentially inappropriate medications (PIMs), such as antipsychotics, benzodiazepines, and hypoglycemics (sulfonylureas and insulin), is more common compared to those without dementia.^3,4^ The use of PIMs is associated with a higher risk of adverse events and outcomes, including falls, worsening cognitive impairment, delirium and confusion, functional decline, and all-cause mortality.^5–9^ Prior studies show that PIM use increases healthcare utilization and costs, with harms far outweighing benefits for patients, payers, and society.^10^ Deprescribing has been conceptualized as a form of de-implementation, in which clinicians reduce or discontinue low-value or harmful practices.^11^ Accountable Care Organizations (ACOs) are an important setting for deprescribing initiatives because they care for substantial populations of older adults, play a central role in coordinating care across providers, and operate under value-based care models that incentivize reducing low-value services.^12^ As such, deprescribing PIMs represents an opportunity for ACOs to improve quality and reduce costs for Medicare beneficiaries. As ACOs continue to pursue value-based care, incorporating deprescribing into primary care settings is a valuable strategy, given primary care’s role as the foundation for high-quality care across the continuum.^13^ By drawing on their long-term relationships, understanding of patients’ life contexts, and interdisciplinary care coordination, primary care clinicians, including physicians, nurse practitioners, and pharmacists, play roles in supporting patients and families over time and coordinating dementia care.^14,15^

Various deprescribing interventions have been designed and implemented among older adults, including those living with dementia.^16–19^ These efforts, such as medication review and educational initiatives targeting patients, care partners, and healthcare providers, have demonstrated improvements in medication-related outcomes. However, their effectiveness on patient-centered clinical outcomes (e.g., falls, other adverse events) remains uncertain. Most deprescribing studies have been conducted in long-term care and inpatient settings, with limited implementation in primary care.^20^ Thus, the current body of evidence provides limited insight into which interventions are effective in deprescribing PIMs for persons with dementia in primary care settings.

Clinical champions are an established strategy for driving uptake of evidence-based practices, ^21,22^ with potential to be effective in efforts to de-implement low-value care. Yet their role in deprescribing PIMs among persons with dementia remains underexplored.^23,24^ We embedded a 24-month pragmatic cluster-randomized clinical trial of a champion-led deprescribing intervention in primary care clinics across two large ACOs (NCT05359679).^25^

Trial sites included two ACO-based systems: Site 1 comprised 46 hospitals and over 370 healthcare centers, with approximately 3% of its Medicare beneficiaries living with dementia; Site 2 was a multi-state home-based care network in which over 20% of Medicare beneficiaries were living with dementia. With leadership support, one clinician per intervention clinic was recruited as a Value Champion to lead deprescribing, and at Site 1, clinical pharmacists served in this role due to primary care capacity constraints.

Details of the intervention and trial design are published elsewhere.^25^ Briefly, the intervention was delivered in two sequential parts. First, champions participated in a 6-month “Value Champions Training Program,” designed to inform, guide, and support champions in carrying out PIM de-prescribing projects at their sites. This training was based on a curriculum developed through a Robert Wood Johnson Foundation Value Champions fellowship program directed by the project’s principal investigator (Supplement A). The training program was conducted through 1-h bi-monthly training webinars. Second, upon completion of the training, each champion received a project workbook to guide their deprescribing work and launched a 12-month deprescribing project at their clinic. During this deprescribing phase, monthly 1-h shared-learning calls open to all champions provided a forum to exchange experiences, learn from one another, and brainstorm solutions.

Guided by the de-implementation outcomes framework, we conducted a qualitative study within the trial to (1) understand clinical champions’ perspectives of their projects and (2) identify key contextual factors that influenced champions’ deprescribing PIMs for persons with dementia in ACO primary care.

## METHODS

### Study Design and Study Participants

This qualitative study was guided by the Prusaczyk et al.^26^ de-implementation outcomes framework, which comprises eight outcomes of stopping low-value or unsafe practices. We focused on fidelity, acceptability, feasibility, and spread, and added equity as another important de-implementation outcome.^27^ We defined deprescribing PIMs for persons with dementia as the clinical practice targeted for de-implementation (i.e., the practice we sought to reduce). The use of trained clinical champions was conceptualized as the key de-implementation strategy, enhanced by training and support through monthly learning calls. Using the de-implementation outcomes framework, we evaluated champions’ perspectives on the success of deprescribing PIMs. Data collection and analysis were conducted between December 2022 and May 2024. The study was approved by Kaiser Permanente’s Washington Institutional Review Board. We report findings according to the Consolidated Criteria for Reporting Qualitative Research (COREQ) guidelines.^28^

All clinician champions from the intervention arm who completed the deprescribing training (*N* = 19) were invited to participate in one-on-one, semi-structured interviews near the end of their 12-month project. Invitations were extended both during monthly learning calls and via individualized email; participants received a $100 honorarium. Although champions’ engagement was high during the training phase, with an average webinar attendance of 94%, it declined during the deprescribing phase, with average participation in shared-learning calls at 36% (Supplement B). Two members of the research team (MP and JP) facilitated the learning calls during the trial, and participants had prior professional contact with them before interview recruitment.

### Data Collection

Data for this study were from two primary sources: transcripts of monthly learning calls and semi-structured interviews with clinician champions. The learning call transcripts captured real-time discussions of challenges and adaptations during implementation, whereas the interviews provided more reflective accounts guided by the interview guide. The 30–60-min interviews were conducted via a secure teleconference platform and co-facilitated by two research team members (MTK, LK, LP, and MP all led interviews) using a guide iteratively co-developed by the team (Supplement C). The guide probed champions’ perspectives on the de-implementation outcomes (appropriateness, feasibility, fidelity, spread, and equity; see definitions in Supplement X), and included questions about barriers and facilitators to outcomes. The interviews and learning calls were held on Microsoft Teams, audio-recorded with participants’ verbal consent and transcribed professionally (interviews) or via the platform’s functionality (learning calls).

A multidisciplinary research team conducted the study. MTK, LK, and LP hold master’s or doctoral degrees with specialized training in qualitative methods; MP, a physician–scientist, served as the original principal investigator (PI), with LP assuming PI responsibilities during the study’s final year. RP and JP contributed expertise in dementia care and ACOs. LK and MP had expertise and prior experience with the RWJF Value Champions model and de-implementation concepts, which informed understanding of the de-implementation context relevant to deprescribing. The research team included four women and two men.

### Qualitative Analysis

We used Atlas ti.^29^ for thematic analysis from a post-positivist perspective.^30,31^ For the semi-structured interviews, we applied a combination of deductive and inductive approaches, while learning call transcripts were analyzed inductively to identify contextual factors. The deductive approach captured champions’ perspectives on de-implementation outcomes, and the inductive approach identified broader contextual influences.

We used a deductive framework analysis^32^ guided by the de-implementation outcomes framework to organize the interview data into predefined domains (e.g., appropriateness, feasibility) and categories (e.g., barriers and facilitators to project feasibility, see Supplementary Tables D and E). MTK and LP developed an initial codebook based on these domains and key interview guide questions. MTK and LP independently coded two transcripts to test the coding structure, refined code definitions iteratively, and met weekly to resolve discrepancies and ensure consistency.

In parallel, we conducted inductive analysis to identify themes that extended beyond the predefined framework. Inductive codes were iteratively developed within each framework domain to capture contextual factors influencing champions’ deprescribing of PIMs, such as caregiver dynamics, system barriers, and clinician capacity. For quality assurance, MTK and LP double-checked approximately 10% of all coded segments across the dataset and reconciled differences through discussion to refine code definitions and ensure consistency. After coding, they developed analytic memos for each domain and compared coded data between non-prescribing clinicians (pharmacists) and prescribing clinicians (physicians and nurse practitioners) to identify overarching and role-specific patterns. During this analysis phase, we focused on appropriateness, feasibility, and fidelity, which were most frequently discussed and most relevant to understanding champions’ implementation experiences given the decline in engagement during the deprescribing phase. Through iterative discussions of the analytic memos, we developed themes that characterized contextual influences on champions’ deprescribing processes. We classified overarching patterns as themes and more specific patterns as subthemes (summarized in Supplementary Table F). We determined that data saturation had been reached when no new insights emerged in subsequent interviews.

## RESULTS

A total of 11 champions (58% of all clinician champions) participated in this qualitative study, including 6 physicians, 2 nurse practitioners (NP), and 3 pharmacists (Table 1). All interview participants attended at least one learning call, although not all learning call participants completed an interview (8 of the 11 had both learning call and interview data).

**Table 1:** Participant Characteristics (*N* = 11)

Participant ID (site-participant no.)	Clinical role
S01-01	Pharmacist
S01-02	Pharmacist
S01-03	Pharmacist
S02-04	Physician
S02-05	Nurse practitioner
S02-06	Nurse practitioner
S02-07	Physician
S02-08	Physician
S02-09	Physician
S02-10	Physician
S02-11	Physician

### Champions’ Perspectives on the De-Implementation Outcomes of the Deprescribing Project

Champions’ perspectives on appropriateness, feasibility, fidelity, spread, and equity of their deprescribing projects are summarized in Table 2.

**Table 2:** Summary of Qualitative Findings on Clinical Champions’ Perspectives on De-implementation Outcomes, Challenges, and Facilitators of Efforts to Deprescribe Potentially Inappropriate Medications (PIMs) for Persons with Dementia in Accountable Care Organization (ACO) Primary Care Settings

De-implementation outcome	Summary of champions’ perspectives	Key challenges	Key facilitators
**Appropriateness**	Champions reported that de-prescribing of PIM was appropriate for their patients in their clinical settings.	Not reported	Not reported
**Feasibility**	Champions reported various inner and outer contextual challenges in executing deprescribing projects, which undermined feasibility.	• High staff turnover • Time constraints and limited opportunities to engage with patients and families • Lack of access to user-friendly data to identify target patients • Organizational changes increasing workload and causing operational disruptions • Difficulty coordinating deprescribing efforts and engaging with colleagues due to varied schedules and workflows • Hesitation from family members and care partners	• Dementia-specific deprescribing training • Support from care partners • Pharmacists served as liaisons and translators across disciplines
**Fidelity**	Champions reported challenges in rolling out their deprescribing projects as planned. Fidelity was affected by competing priorities, shifting leadership expectations, external disruptions, and the need for sustained team engagement and trust-building.	• Delays due to the COVID-19 pandemic and a hurricane • Competing priorities • External disruptions (busy quarters, external factors) • Lack of leadership and team buy-in • Inconsistent ability to track and monitor progress	Not reported
**Spread**	Most champions did not report formal spread of deprescribing efforts across clinical settings. However, informal collaborations, success stories, leadership support, and future recommendations (e.g., dashboards, handouts) may facilitate future spread. Some champions described growing cross-clinical interest generated through their initial deprescribing work.	• Collaborative dynamics required negotiation of priorities across teams • Lack of formal pathways to spread practices across settings • Need for data visualization to support buy-in • Hesitance among some teams to adopt deprescribing without seeing clear success stories	• Leadership support • Success stories and peer learning • Interest from additional clinical areas once initial value was demonstrated
**Equity**	Champions did not report specific inequitable outcomes of deprescribing. However, broader community and social inequities emerged as important factors affecting deprescribing success. Champions recognized that patients’ and care partners’ social resources, particularly time, education, and financial means, contributed to inequities in access to and engagement with deprescribing efforts. Champions identified resources to support connections with patients and families as facilitators.	• Care partners’ availability and capacity influenced which patients could participate in deprescribing, potentially favoring more affluent patients with greater family support • Patients and care partners from lower socioeconomic backgrounds faced greater barriers to attending clinic visits and managing care tasks	• Partnerships with community resources (e.g., meal programs, care partner support programs, Medicaid waiver services) • Easily accessible resource lists to connect families with available supports

#### Appropriateness

All champions reported that deprescribing PIMs was appropriate for their patient populations and clinical contexts. They viewed deprescribing PIMs as an important component of high-value care. As one champion shared:

“I’d need a stronger word than absolutely. Yes, I think this is definitely a good fit for our patient population. We see a lot of patients who are homebound, with mobility limitations, fall risk, dementia, so it’s definitely appropriate.”—S02-07-Physician.

At the same time, champions acknowledged that appropriateness is not one-size-fits-all, and providers need to make individualized decisions about what is appropriate in each case. As a champion noted:

“But make sure that they realize too that it’s up to their discretion as providers to what’s appropriate for patients as well. Because of course it’s not a blanket, right? Not everything is appropriate for every scenario”—S02-06-NP.

#### Feasibility

While the concept of deprescribing PIMs was appropriate, champions reported multitudes of barriers to the feasibility of their deprescribing projects, as expressed by a champion who said, “I feel it’s very appropriate. Is it difficult? Yes…”—S02-05-NP. The feasibility of these projects was undermined by a complex interplay of multi-level challenges, including but not limited to organizational-level barriers (e.g., staff turnover) and process-level barriers (e.g., difficulties in coordinating deprescribing with care team members), as one champion described:

“Unfortunately [things are] not going so good, especially talking with the colleagues because there are a lot of changes happening in the practice and everybody is already overwhelmed with those changes”—S02-07-Physician.

Champions identified several facilitators that supported their deprescribing projects, including the content of dementia training, support from care partners, and pharmacists’ organizational role, which enabled them to serve as liaisons to foster collaboration among stakeholders. These facilitators were frequently enhanced through relationships and communication. This highlights the value of the training the champions received, which equipped them with knowledge about PIMs and strategies for engaging diverse stakeholders (Supplement A). As one champion described:

“I think [the training] helped us … I was not fully aware of how to progress with it or how to talk to my patients about it or show some clinical data or something. But now since I have seen all those videos and spoken to all those people, it has kind of reinforced what we usually do. It was very helpful. With all those things that we learned, I think that has kind of given me the confidence now to talk about this thing in a much better way than previously that I would have thought.”—S02-09-Physician

#### Fidelity

Champions reported that the fidelity of their deprescribing projects and ability to perform their role as planned were severely limited. Organizational and other factors, including COVID-19, made it difficult to secure leadership and team buy-in and to sustain team engagement and relationship-building, as colleagues’ attention and priorities were directed elsewhere. As one champion described:

“At the heat of COVID, during [hurricane] Delta, during Omicron surges, we did not have enough healthcare practitioners, so outpatient clinicians were being deployed on the inpatient setting to help support acute care and everybody was tired and burnout was a concern…. So to meet with the physician leaders to say ‘hey, we think this is a patient care quality opportunity’, you get a lot of ‘let’s talk about this again in a month’”—S01-04-Pharmacist.

#### Spread

Most champions did not report formal spread of deprescribing practices beyond their immediate clinical settings. However, informal exchanges and emerging success stories fostered early interest in broader dissemination, despite the lack of formal pathways. As one champion noted:

“Well, it started with dementia, and the heads of the neurology team saw the value and said we need help over here too. You’re good at this, we also need your help here and here.”—S01-01-Pharmacist

#### Equity

Champions also expressed awareness of equity concerns, recognizing that whether patients have family resources may shape the ways in which they benefit from deprescribing efforts:

“… whether the family members have the time or the support to bring these patients in for multiple visits per year, if there was an equity issue, that is how I would identify it.”—S01-04-Pharmacist.

Champions attempted to adapt their approaches to promote equity in deprescribing through individualized care, partnerships with community services, and persistent follow-up. As one champion shared:

“I will say that thinking about ways that you can also help patients to connect to resources…. I know if somebody has an acute illness, there’s certain services that are available. But a lot of people … don’t know where to find the resources.”—S02-10-Physician

### Key Contextual Factors Shaping Champions’ Deprescribing Efforts

**Figure 1:**
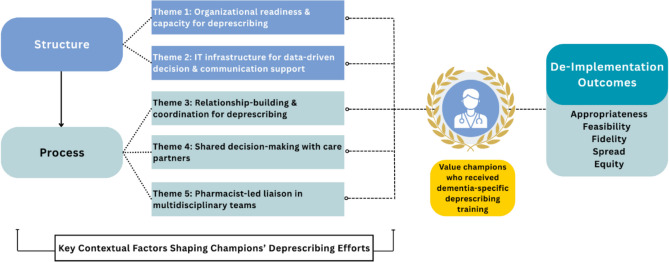
Champion-reported conceptual model of key contextual factors shaping Champions’ deprescribing efforts and de-implementation outcomes

In examining the contextual factors influencing champions’ experiences across all five de-implementation outcomes, we identified five key themes: (1) organizational readiness and capacity for deprescribing; (2) data-driven decision and communication support; (3) relationship-building and coordination for deprescribing; (4) shared decision-making with care partners; and (5) pharmacist-led liaison in multidisciplinary teams (Fig. 1).

**Figure 2:**
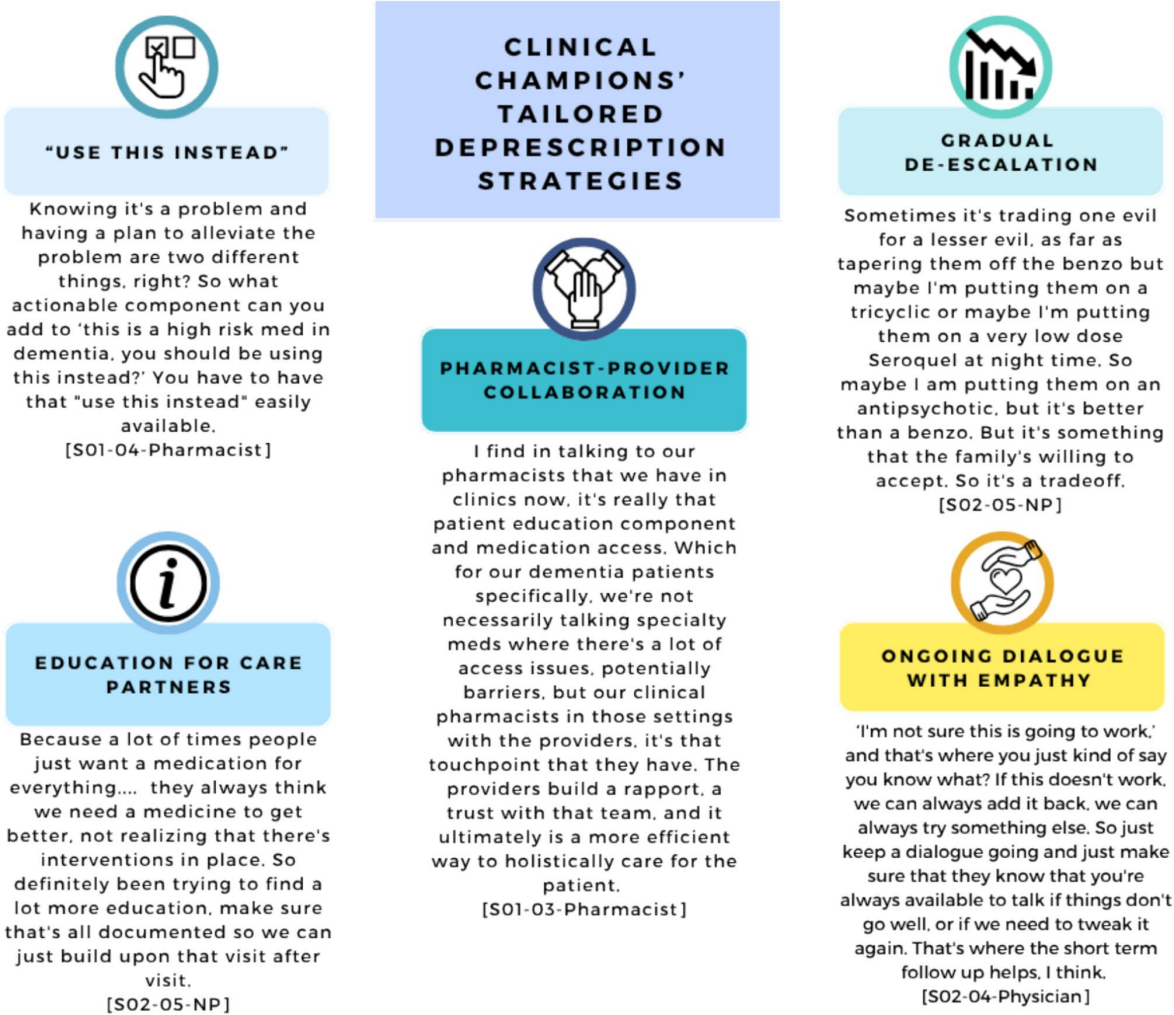
Illustrative quotes for clinical champions’ tailored deprescribing strategies

#### Theme 1: Organizational Readiness and Capacity for Deprescribing

Champions described how external events and shifting organizational priorities created significant challenges for their deprescribing projects. At Site 1, several clinics closed during the intervention period due to a Category 4 hurricane, resulting in the reassignment of primary care patients to contracted providers outside the ACO’s internal delivery system. That sudden surge in patient volumes, combined with healthcare worker burnout and high turnover, further complicated deprescribing efforts:

“We were supposed to do this (deprescribing intervention) in February 2022 …. and I was told no, Delta’s killing us, now is not a good time… We came back in April-May and they were like we’re just catching our breath. We’re doing some other things, just wait. And then I came back in June-July, and they’re like, Omicron’s killing us now.”—S01-04-Pharmacist

Staff turnover was a persistent barrier, as champions often had to restart deprescribing conversations whenever engaged colleagues left the organization:

“We’ve got a lot of turnovers going on right now, it’s hard to keep people. So we’re all just kind of doing the best we can to keep our heads above water at this point in time.”—S02-06-NP

At Site 2, market-driven organizational restructuring and rebranding introduced a new computer system, revised work schedules, and a newly appointed leadership team. Champions had to continually adapt, making it difficult to launch and sustain deprescribing activities:

“They’ve implemented a lot of new things here…. so kind of just going through that, like our scheduling has changed, our routes aren’t quite the same as they used to be.”—S02-06-NP

#### Theme 2: Information Technology (IT) Infrastructure for Data-Driven Decision and Communication Support

Champions emphasized that user-friendly data tools are needed to support the identification of target patients for deprescribing. However, limited access to such data posed a significant barrier:

“I’d like for us to have in our office easy access for data…. I want a list of people with dementia that are being prescribed these medications. What I’m being given is a spreadsheet with many intricate moving parts…. It’s very much a data analyst driven tool. It’s not what I want to see. I want to see a list…. So, it’s basically falling back on me to manage all of that data myself, and I don’t have the time to do that. I’m not a data analyst, I’m a nurse practitioner, I take care of people”—S02-05-NP.

Another champion also shared:

“My challenge was to review the chart, getting the data correct because I was not happy with getting data from our institution - are those patients currently on the medications and if they are, are they getting it from us or from other providers or what’s going on?”—S02-04-Physician

Access to patient data could have allowed champions to systematically review medication lists and engage prescribing providers in deprescribing efforts. Champions also viewed data as a strategic tool to build project momentum and foster collaboration across the care team. As one champion noted:

“… this project has really helped to understand the language of the data, how to communicate it effectively… it puts a framework around what story can we tell to continue the momentum on the model that we’re setting up to build these interdisciplinary teams and take better care of the patients.”—S01-03-Pharmacist

In practice, however, limited access and the time-consuming process of identifying target patients made it challenging to incorporate deprescribing into routine care, ultimately undermining the intervention’s feasibility.

#### Theme 3: Relationship-Building and Coordination for Deprescribing

Champions reported that successful deprescribing depended on strong relationships and coordination among providers. They described how fostering collaboration across the care team was essential to implementing and sustaining deprescribing efforts. However, relationship-building and coordination demand considerable effort and time for frequent communication, which champions found challenging given time constraints. As one champion shared:

“It was really hard for me to try and discontinue the insulins because I found that once I discontinued it, sometimes some of my colleagues restarted them…. because I really haven’t had a chance to communicate well with my colleagues about the purpose of our study…. A lot of times we just don’t have time to go back and read everybody’s notes about what you did. So there’s a lot of communication that doesn’t happen and there’s really no time to go up to somebody and say hey, listen, I’m going to go see your patient – can you give me a rundown about what’s going on with them? There’s really no time for that communication.”—S02-05-NP.

#### Theme 4: Shared Decision-Making with Care Partners

Deprescribing for persons with dementia is inherently dyadic, as decisions are shaped not only by clinicians but also by care partners—whether they are employed caregivers in long-term care settings or family members—who support day-to-day medication management. As one champion noted:

“… in dementia specifically, you’re never just treating the patient, you’re also treating the patient’s caregiver.”—S01-01-Pharmacist.

Deprescribing discussions needed to address not only the potential benefits of deprescribing PIMs for patients, but also the implications for care partners, who may view these medications as helpful in managing caregiving challenges:

“Everybody wants a pill, everybody wants an easy fix, and sometimes there just aren’t easy fixes. I explain that to people too. A pill isn’t going to fix this situation, and I can try to make things a little bit easier for you, but a benzo isn’t going to make this an easy fix either, so we need to find the safest option for your loved one.”—S02-06-NP

Champions tailored conversations by listening carefully to care partners’ concerns. Multiple deprescribing conversations could be required as champions frequently encountered resistance from care partners who were reluctant to engage with the idea of reducing or renouncing certain medications:

“You just have to kind of know each family and each caregiver…. There was one patient who we really did have a lot of success. She had been on Lorazepam, and we were finding that Buspirone was working better. So over a number of visits, speaking with the staff, speaking with the daughter, we got her a little more Buspirone, very minimal Lorazepam. But it took a lot of work and a lot of tracking people down. I think it was an over the time thing.”—S02-07-Physician

#### Theme 5: Pharmacist-Led Liaison in Multidisciplinary Teams

Pharmacist champions’ experiences were distinct from those of physician and nurse practitioner champions due to their roles as non-prescribing clinicians. They viewed their role as providing pharmacy expertise to inform physicians’ deprescribing decisions and strategically engaging physicians and nurses to build support for deprescribing initiatives. As a result, much of their effort focused on building trust and collaborative relationships:

“Really having to focus my efforts on being this liaison and translator for nursing, physicians, pharmacy, and IT, but also for vendors that are working with us now. I have to speak all of their languages”—S01-02-Pharmacist.

Pharmacist champions leveraged their strengths as liaisons to facilitate communication among providers, patients, and families. They supported providers in navigating the complexities of medication modifications. As one pharmacist champion described:

“One of the things that makes us interesting is we are a pharmacist cohort, and so discussions pharmacist to physician versus physician to physician are a little bit different… I usually try to approach physician to pharmacist conversations as: a) seeking to understand, and then b) I will attempt to find a physician champion and get them to make recommendations to their colleagues and their partners, versus trying to give my own recommendations”—S01-01-Pharmacist.

Importantly, champions used adaptive, relationship-centered strategies to navigate deprescribing in their local contexts (Fig. 2). These included suggesting alternative medications, using resources to address colleagues’ concerns, and educating patients and families about medication safety. As one champion explained:

“Knowing it’s a problem and having a plan to alleviate the problem are two different things. You need to make alternatives easily available”—S01-01-Pharmacist.

Champions also emphasized the importance of skills to foster ongoing, empathic dialogue with patients and families. As one champion shared:

“If families and caregivers don’t feel left on their own, they are more receptive to de-escalating medications. People often expect a pill, but not everything is solved by a pill”—S02-04-Physicians.

## DISCUSSION

This study explored champions’ perspectives on the outcomes of deprescribing of PIMs for persons with dementia within ACO primary care settings. The study took place amid significant disruptions, including organizational restructuring, the COVID-19 pandemic, and natural disasters, all of which influenced the implementation phase of the intervention. Champions reported that the feasibility and fidelity of their deprescribing projects were largely undermined by these external and organizational challenges. We identified five key structural and process-level factors that shaped champions’ deprescribing experiences. Structural factors included (1) external influences that affected organizational readiness and capacity to support deprescribing efforts, and (2) inadequate IT infrastructure, which limited access to data needed for identifying target patients and communicating with colleagues. Process-level factors included (3) the importance of relationship-building and care coordination in facilitating deprescribing, (4) the dyadic nature of deprescribing for persons with dementia, which required shared decision-making with care partners, and (5) pharmacists’ specialized medication knowledge and role as liaisons within multidisciplinary teams to encourage collaborative deprescribing.

Our findings complement prior studies demonstrating that deprescribing is a multifaceted, context-specific process shaped by external and internal influences.^33–36^ Prior studies have shown that clinical champions can help overcome barriers and facilitate cultural change; however, a champion-led strategy alone is insufficient to ensure successful deprescribing.^24,37–39^ In this trial, champions received dementia-specific deprescribing training prior to implementation and participated in monthly shared learning calls for ongoing support, ensuring that the intervention did not rely solely on individual champions’ commitment and motivation. Champions’ experiences also reflected that their ability to enact deprescribing depended on the interdependent relationships among multilevel contextual factors. Clinical champions highly valued the training (Supplement A), which equipped them with practical strategies they could use. They attempted to translate this knowledge into action by tailoring deprescribing efforts to their local contexts. Although these trained champions faced numerous barriers to deprescribing, they were able to apply what they learned to navigate deprescribing conversations with colleagues and care partners. These findings suggest that incorporating deprescribing content into annual continuing education may help cultivate a clinical culture that facilitates deprescribing PIMs as a key component of dementia care. Additionally, pharmacist champions in our study facilitated collaborative deprescribing among providers, patients, and care partners. As such, future interventions may benefit from including pharmacists as clinician champions to strengthen care coordination and deprescribing education.^40,41^

Clinical champions’ experiences highlighted key structural and process-related factors influencing champion-led deprescribing efforts. Whereas various interventions have targeted specific contextual barriers, such as enhancing provider knowledge through education, leveraging clinical decision support tools embedded in electronic health records, and implementing multidisciplinary medication reviews, ^20,42–46^ there remains a lack of evidence-based, structured care pathways in dementia that incorporate deprescribing of PIMs.^47^ Comprehensive Geriatric Assessment (CGA), a multidisciplinary model originally developed for inpatient settings, has more recently been adapted for use in primary care.^48^ Although not specific to dementia, CGA includes systematic medication review and could be adapted to better support deprescribing within the context of dementia care. Altogether, our study findings suggest that while a champion-led strategy can effectively address individual-level factors (e.g., provider and care-partner knowledge and attitudes toward PIMs), it is not designed to overcome structural barriers such as insufficient organizational readiness or IT infrastructure.

ACO primary care clinician champions’ experiences highlighted the promise of value-based settings for deprescribing. Champions emphasized that long-standing relationships with patients and care partners allowed for iterative dose reductions and ongoing deprescribing conversations. This relational continuity, supported by the structure of the ACO primary care model, presents a promising avenue for deprescribing PIM.

Consistent with prior work, primary care clinical champions in our study leveraged their central role as the main point of contact for persons with dementia and their care partners.^49^ Their holistic understanding of patients’ medical and life histories positioned them to lead a patient- and family-centered deprescribing process. At the same time, system-level challenges persist, including workforce turnover and limited health information technology capacity to generate data that are actionable at the point of care. Strengthening deprescribing in ACO primary care could benefit from explicitly embedding PIM-reduction goals into organizational structures (e.g., by creating and displaying data dashboards) and linking them to financial incentives that acknowledge the additional time and relational work required for deprescribing in dementia care.

Our study underscores the dyadic nature of deprescribing for persons with dementia, as both paid and unpaid care partners actively engage in discussions and decisions about medications, adding evidence to the current body of work demonstrating that caregiver factors influence the use of PIMs.^50^ Niznik et al. conceptualized deprescribing as an integral element of goal-concordant dementia care, highlighting its role in aligning medication management with the evolving goals and values of patients and their care partners.^51^ These insights on the critical role of caregiver involvement and a goal- and value-centered approach to deprescribing can be reinforced by value-based payment models such as the Centers for Medicare & Medicaid Services (CMS) GUIDE (Guiding an Improved Dementia Experience) Model.^52^ The GUIDE Model recognizes care partners as essential members of the care team and promotes more comprehensive, person- and care partner-centered dementia care. Future studies exploring how to align deprescribing interventions with value-based payment incentives and quality metrics may offer an important opportunity to support safer, and more tailored medication management within dementia care workflows. ^53,54^

Our study has limitations. It was unclear whether those nominated by ACO leadership to be champions were genuinely motivated or interested in the role. Champions who did not participate in any learning calls were also invited to the study, but none enrolled, which may limit our ability to fully characterize the experiences of the least engaged champions. Our study gathered champions’ perspectives on the specific skills needed to be an effective champion, but did not measure these skills directly, limiting understanding of a possible causal relationship to success.^24^ Only a subsample of champions participated in interviews or attended most learning calls, reflecting a decline in engagement during the deprescribing phase (Supplement B). This could be a source of bias, as the most successful or motivated champions likely participated. We did not collect the champions’ demographic information that might have provided additional context to their reported experience.

## CONCLUSIONS

This study identified key contextual factors that shape champion-led deprescribing for persons with dementia in ACO primary care settings. Dementia-specific deprescribing training equipped champions with practical strategies to tailor their efforts to local contexts. Primary care clinicians are uniquely positioned to lead personalized deprescribing conversations, while pharmacists can play an important role by liaising with providers, patients, and care partners to coordinate deprescribing. The findings demonstrated that effective champion-led deprescribing efforts require supportive organizational structures, including ongoing education, accessible data for clinical decision support, and multidisciplinary collaboration. Future deprescribing interventions can leverage the principles of value-based care and payment models to strengthen system-level coordination for deprescribing PIMs and promote safer medication management in dementia care.

## Supplementary Information

Below is the link to the electronic supplementary material.

ESM1 (PDF 466 KB)

## Data Availability

Data from this study will not be publicly available due to confidentiality and privacy reasons. All data reported within the manuscript have been anonymized and de-identified.
