# Supplementary material for: Champion-Led Deprescribing for Persons with Dementia in Primary Care: A Qualitative Study in Accountable Care Organizations
Source: J Gen Intern Med. 2026 Feb 2;41(10):2844–55. doi: 10.1007/s11606-026-10234-8 (PMC12951280; doi:10.1007/s11606-026-10234-8)
Supplement: Supplementary file 1 — (PDF 466 KB) [file 11606_2026_10234_MOESM1_ESM.pdf]

Supplementary Materials

Champion-led Deprescribing for Persons with Dementia in Primary Care: A Qualitative Study in Accountable Care Organizations

Masami Tabata-Kelly et al.

Contents

Supplement A: Value Champion Learning Modules 2
Supplement B: Attendance rates for training webinars and learning calls 3
Supplement C: List of semi-structured interview questions 4
Supplement D: Definitions of De-implementation Outcomes[1] 4
Supplement E: De-implementation Outcomes Framework domains and associated categories 5
Supplement F: Themes and sub-themes 9
Supplement G: Consolidated criteria for reporting qualitative studies (COREQ): 32-item checklist 14

Supplement A: Value Champion Learning Modules

- Module 1: High value care and health equity
- Module 2: Engaging leadership for high value care
- Module 3: Choosing an overused service
- Module 4: How to conduct a stakeholder assessment
- Module 5: Engaging patient participation in your project
- Module 6: Measurement, data and trust supporting change with data
- Module 7: Engaging health care professionals
- Module 8: Strategies employed by value champions
- Module 9: Choice architecture and overuse reduction
- Module 10: Planning for sustainment

Supplement B: Attendance rates for training webinars and learning calls

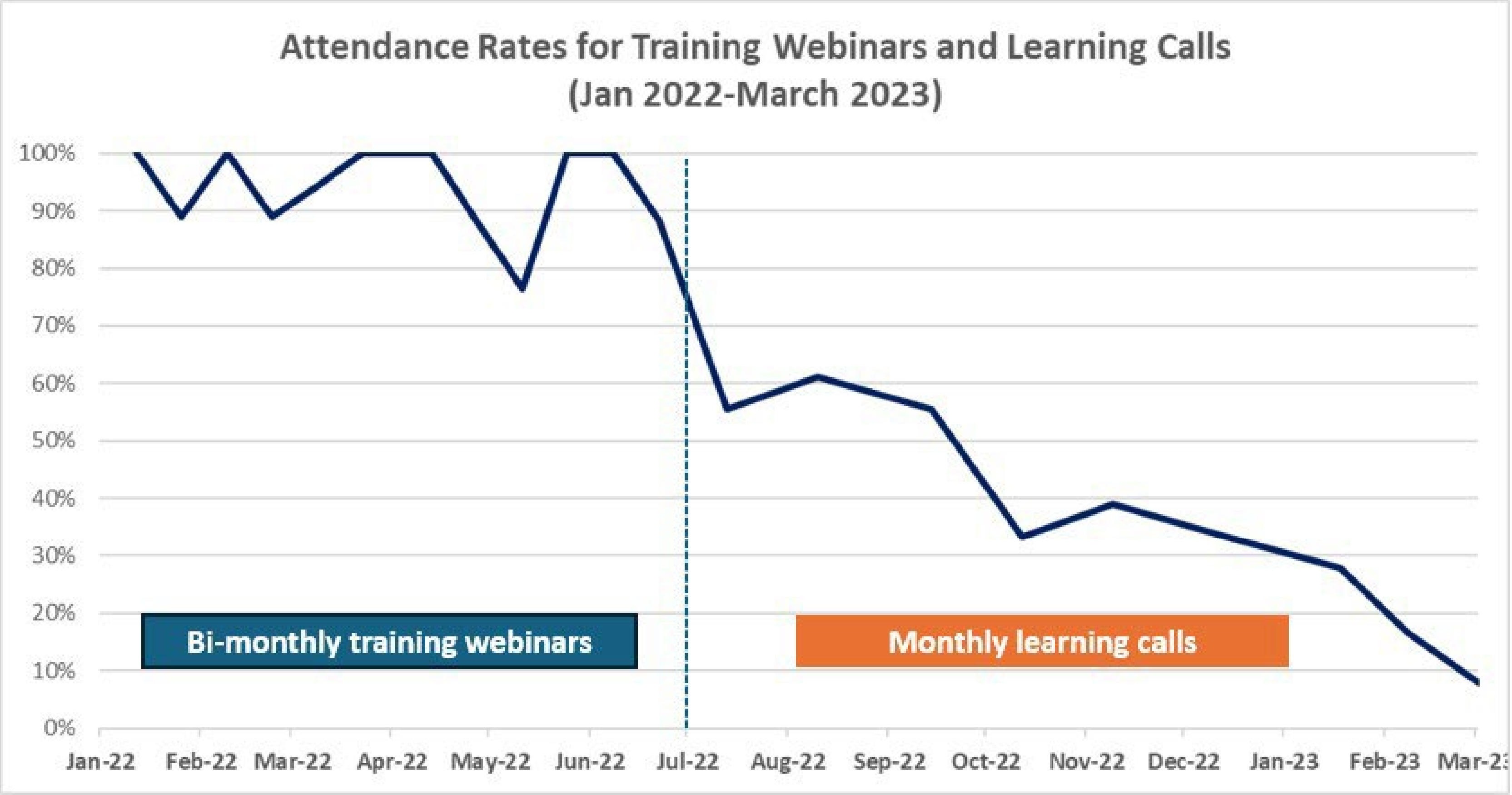

Supplement C: List of semi-structured interview questions

Domain	Interview questions
**Clinician background**	• Clinical role<br>• Practice setting<br>o How long have you been in your organization?<br>o How often do you see a patient with dementia<br>o How many are on one of these meds (anti-psychotics, benzodiazepines, or hypoglycemic medications) ?
**Overall experience as a clinician champion**	• Can you tell us about your overall experience thus far of being a clinician champion for this project in your organization?<br>• What class/group of potentially inappropriate medications did you prioritize to work on and why? (anti-psychotics, benzodiazepines, or hypoglycemic medications)
**Your perspectives as a clinician champions - Appropriateness**	• **Appropriateness[1]:** Is it a good idea? Does it make sense to do this with your patients in your clinician setting?<br>• Appropriateness in your clinical setting?<br>• From the patient and family's perspectives?<br>• Perspectives from your clinical colleagues?
**Your perspectives as a clinician champion - Feasibility**	• **Feasibility[1]:** Is it possible? Is it do-able? For example, it might be a good idea, but it may be more or less feasible for a variety of reasons.<br>• How feasible was it for you to:<br>○ Decrease use of potentially inappropriate medications in your patients?<br>○ Be an advocate/champion for this project?<br>• What made it less feasible?<br>• Resources/support that could have made it more feasible
**Your perspectives as a clinician champion - Fidelity**	• **Fidelity[1]:** Were you able to roll out your plan and implement as planned?<br>• With your own patients? Why or why not?<br>• To encourage your clinical colleagues to decrease the use of these meds with their patients? Why or why not?<br>• If you did change your plan, what happened that caused this change?

**Your perspectives as a clinician champions - Spread**	• **Spread[1]:** How well efforts to decrease the use of these meds have spread across the clinical setting where you work<br>• How much success have you had in reaching out to all providers in your clinical setting about decreasing the use of these meds?<br>• Have you seen or heard of anything that would indicate that individuals who you have not had direct contact with are aware of efforts to decrease the use of these medications?<br>• Have you thought about how to facilitate/support efforts to spread decreased use of these meds beyond your own clinical setting?
**Your perspectives as a clinician champion - Equity**	• **Equity[1]:** Sometimes when we try to make a change or improvement in clinical care, some patients might benefit more, or have a higher risk of harm, than others due to a characteristic such as race/ethnicity, income or education.<br>• What are your thoughts on the equity aspect of this project?<br>• If any, could you share any challenges related to equity in implementing this project that you or your colleagues have experienced?<br>• Did patients or their family/caregivers mention concerns about discrimination or unequal treatment when efforts were made to decrease the use of these meds?

[1]Definitions of De-implementation Outcomes based on the following sources:

- Prusaczyk B, Swindle T, Curran G. Defining and conceptualizing outcomes for de-implementation: key distinctions from implementation outcomes. Implement Sci Commun. 2020;1(1):43. doi:10.1186/s43058-020-00035-3.
- Proctor EK, Bunger AC, Lengnick-Hall R, et al. Ten years of implementation outcomes research: a scoping review. Implement Sci. 2023;18(1):31.

Supplement D: Definitions of De-implementation Outcomes[1]

De-implementation outcome	Definition
**Appropriateness**	The degree to which stopping a practice or the idea of stopping a practice is perceived to not fit, have relevance, or be compatible for a given setting, provider, consumer, issue, or problem.
**Feasibility**	The extent to which a practice can be successfully stopped within a given agency or setting
**Fidelity**	The degree to which the practice is stopped for the right people and in the right contexts; **or** the degree to which the practice is stopped equally, across patients/clients and providers; **or** the degree to which the clinician champion intervention was implemented as it was prescribed in the original protocol or as it was intended by the program developers
**Spread**	The extent to which the practice is discontinued within a service setting and its subsystems
**Equity**	The degree to which stopping the practice aims to reduce and eventually eliminate disparities in health and in the determinants of health that adversely affect excluded or marginalized groups; or the proactive tailoring of de-implementation strategies to address healthcare inequities and unintended consequences for vulnerable populations.

[1]Definitions of De-implementation Outcomes based on the following sources:

- Prusaczyk B, Swindle T, Curran G. Defining and conceptualizing outcomes for de-implementation: key distinctions from implementation outcomes. Implement Sci Commun. 2020;1(1):43. doi:10.1186/s43058-020-00035-3.
- Proctor EK, Bunger AC, Lengnick-Hall R, et al. Ten years of implementation outcomes research: a scoping review. Implement Sci. 2023;18(1):31.

Supplement E: De-implementation Outcomes Framework domains and associated categories

De-implementation framework domain	Category	Description
**Appropriateness**	General	Use this code when champion described their overall perspectives on the appropriateness of reducing the use of potentially inappropriate medications (PIMs).
	Patients	Use this code when champions reported their thoughts on the appropriateness of decreasing the use of PIMs for patients.
	Patient and family's perspectives	Use this code when champions reported their thoughts on the appropriateness of decreasing the use of PIMs from patients' and families' perspectives.
	Talking to colleagues	Use this code when champions reported their thoughts on the appropriateness of talking with their colleagues about decreasing the use of PIMs.
	Colleagues' perspectives	Use this code when champions reported how their colleagues responded to decreasing the use of PIMs.
**Feasibility**	General	Use this code when champions reported their overall thoughts on the feasibility of decreasing the use of PIMs
	Decrease use of PIM – challenges	Use this code when champions reported factors that made it less feasible to decrease the use of PIMs in their patients with dementia.
	Decrease use of PIM – facilitators	Use this code when champions reported factors that facilitated their ability

De-implementation framework domain	Category	Description
		to decrease the use of PIMs.
	Decrease use of PIM – mitigations	Use this code when champions reported actions they took to overcome challenges related to decreasing the use of PIMs.
	Decrease use of PIM – recommendations	Use this code when champions reported recommendations for improving the feasibility of decreasing the use of PIMs.
	Role as a champion	Use this code when champions reported their experiences serving as champions for decreasing the use of PIMs with colleagues.
	Role as a champion – challenges	Use this code when champions reported challenges to being champions for decreasing the use of PIMs.
	Role as a champion – facilitators	Use this code when champions reported factors that facilitated their ability to serve as champions for decreasing the use of PIMs.
	Role as a champion – mitigations	Use this code when champions reported actions they took to overcome challenges as a champion for deprescribing.
	Role as a champion – recommendations	Use this code when champions reported recommendations for supporting future champions' ability to facilitate deprescribing.
**Fidelity**	General	Use this code when champions reported whether they were able to roll out their deprescribing plan as intended.
	Role as a champion	Use this code when champions reported their

De-implementation framework domain	Category	Description
		experiences talking with their colleagues to decrease the use of PIMs as planned.
	Role as a champion – challenges	Use this code when champions reported challenges related to serving in their role as a champion according to the initial plan.
	Role as a champion – mitigations	Use this code when champions reported actions they took to address challenges related to fidelity in their champion role.
**Spread**	General	Use this code when champions reported their general thoughts on how deprescribing efforts spread within their clinical setting.
	Challenges	Use this code when champions reported barriers to spreading deprescribing practices within or beyond their own setting.
	Mitigations	Use this code when champions reported how they overcame barriers to spreading deprescribing efforts
	Facilitators	Use this code when champions reported facilitators that supported the spread of deprescribing efforts.
**Equity**	General	Use this code when champions reported their thoughts on equity considerations or described challenges related to implementing deprescribing equitably.
	Unequal benefits	Use this code when champions reported that some patients benefited more than others from deprescribing efforts and described who and why.

De-implementation framework domain	Category	Description
	Unequal efforts	Use this code when champions reported differences in the level of effort to decrease PIMs across patient populations or clinical contexts.
	Patient or family concerns	Use this code when champions reported that patients or family members expressed concerns about discrimination or unequal treatment related to deprescribing.

Supplement F: Themes and sub-themes

Theme	Subtheme	Illustrative quote
**Theme 1: Organizational readiness & capacity for deprescribing**	COVID-19 Pandemic	• We were supposed to do this in February 2022, so I started the conversations with [NAME] and … leaders in February, and I was told no, Delta's killing us, now is not a good time. And so we came back in April-May and they were like we're just catching our breath. We're doing some other things, just waiting. And then I came back in June-July, and they're like, Omicron's killing us now. [S01-04-Pharmacist]
	High turnover	• They have had a lot of turnovers…. change management has never been more important because of the speed that change is occurring, but I've never seen turnover like this in my career at the patient facing level, between the front desk staff … the nurses and even the nurse practitioners – the turnover has been unreal…. Unfortunately, one of the things that I've learned is, as you engage the team and engage the nurse practitioners and the RN's – we try to get into patient facing colleagues first – you engage them, you train them, we provide the education, and then they leave. [S01-04-Pharmacist]<br>• Yeah, we've got a lot of turnovers going on right now, it's hard to keep people. So we're all just kind of doing the best we can to keep our heads above water at this point in time. [S02-06-NP]
	Organizational changes	• Unfortunately, not so good, especially talking with the colleagues because there are a lot of changes happening in the practice and everybody is already overwhelmed with those changes. So, I have not had any chance to talk with my colleagues. [S02-08-Physician]
**Theme 2: Information technology (IT) infrastructure for data-driven decision and communication support**	Access to patient lists	• I haven't seen a list for a while and it's just the Excel sheet that prints off all the patients and then you have to put all the pages together. So I haven't done anything. They're all the other providers' patients, so I don't know. How did you get your practice manager to get yours all weaned down to whatever you like? Because no one here will help (laughs). [S02-09-Physician]<br>• My challenge was to review the chart, getting the data correct… are those patients currently on the medications and if they are, are they getting it from us or from other providers or what's going on? At that time, I did not have time to go through the individual chart to look at the details. [S02-08-Physician]

Theme	Subtheme	Illustrative quote
	Actionable data	• I guess the most difficult thing, and I keep kind of going back to this, is the time. Honestly, I thought by now I would have more of my numbers where I wanted to kind of have it tracked, because they are sending us the monthly, which I have saved. It's just a matter of getting the time to go through that. I would say the biggest thing with me is my plan isn't going as planned because I was kind of hoping to already be closer to where we are now in the timeframe of things. So I guess the plan changes now to get caught up and then to keep more of a rigorous schedule being put on track. [S02-06-NP]<br>• I think the data and reporting and having dashboarding will provide visibility to providers as to where their opportunities are. [S02-08-Physician]
**Theme 3: Relationship-building & coordination for deprescribing**	Time constraints	• A lot of times we just don't have time to go back and read everybody's notes about what you did. So there's a lot of communication that doesn't happen and there's really no time to go up to somebody and say hey, listen, I'm going to go see your patient – can you give me a rundown about what's going on with them? There's really not time for that communication. [S02-05-NP]<br>• More time. It's definitely been a high-quality experience, but that's been the big thing is more time. I think that's a challenge in many quarters. [S02-04-Physician]
	Informal conversations	• But I think passing on the success stories has been helpful. Once I'm able to transition from the non-formal conversations to something more formal and be able to show data and be able to visualize where things are, that's going to help a lot more because I know myself, I'm more a visual learner, where you're able to kind of see it. It just makes it easier to kind of retain that, it kind of hits home more, and I feel that would help them as well. [S02-06-NP]
**Theme 4: Shared decision-making with care partners**	Resistance from family care partners	• I'm seeing someone with a family of medical professionals, and she is getting a dose of Ativan (Lorazepam) in the morning, a dose of Ativan in the afternoon, a dose of Ativan at night, a higher dose at night to help her sleep, and then she has it as needed throughout the day. And I was very shocked that her previous primary physician was prescribing these medications. Her gait's not the best, and I tried to approach the subject with the

Theme	Subtheme	Illustrative quote
		family, and as I mentioned, I got shut down pretty quickly…. So that's been challenging. [S02-06-NP]<br>• Every now and again you'll have a family member who's like no, no, that's the pill that makes it so that Mommy gets two hours to herself. Then the conversation takes a little bit of a turn, and at that point we'll usually escalate so that it's the provider, the pharmacist, the family members, and then you have to kind of change the conversation because now you know you're dealing with a different beast. [S01-03-Pharmacist]<br>• Most of the time it is more the caregivers and facilities or family that you're dealing with. The patient usually doesn't have as much objection, and a lot of times what you get – the way they always say it is "Doc, you've got to do something," …. So it always comes to me that they just want something, and that's where you take that time to educate. I think it's a case-by-case basis. Some just want that pill to just fix it. Some are more "I hear what you're saying and I want to do everything I can before we resort to medication." You just have to kind of know each family and each caregiver. And frankly, there are some logistical problems with everybody being pulled in different directions, even finding those people to talk to them. There's just dynamics. [S02-04-Physician]
**Theme 5: Pharmacist-led liaison in multidisciplinary teams**	Pharmacist – provider collaboration	• I find in talking to our pharmacists that we have in clinics now, it's really that patient education component and medication access. Which for our dementia patients specifically, we're not necessarily talking specialty meds where there's a lot of access issues, potentially barriers, but our clinical pharmacists in those settings with the providers, it's that touchpoint that they have. The providers build a rapport, a trust with that team, and it ultimately is a more efficient way to holistically care for the patient. [S01-03-Pharmacist]<br>• I usually try to approach physician to pharmacist conversations is a) seeking to understand, and then b) I will attempt to find a physician champion and get them to make recommendations to their colleagues and their partners, versus trying to give my own recommendations. So you and I have discussed

Theme	Subtheme	Illustrative quote
		this. I usually like to have a soft touch from a change management standpoint, I find that with a physician that goes over well. It started out almost as a conversation, meeting up for lunch to discuss clinical cases, and then kind of letting the conversation progress. [S01-01-Pharmacist]
	Unique challenges as a pharmacist – serving as a "liaison"	• Going from an embedded pharmacist, I was able to say here are optimizations and I can prove my value to physicians…. And so really having to focus my efforts on being this liaison and translator for nursing physicians, pharmacy and IT, but also for vendors that are working with us now. So I have to speak all of their languages. Coordinate training for at every level. [S01-02-Pharmacist]
**Theme 6: Clinical champions' tailored strategies to mitigate deprescribing discussion challenges**	"Use this instead" / Gradual de-escalation	• If families and care givers don't feel like they're left to themselves to deal with this, I think it helps them to be more receptive to deescalating the meds to the non-pharmacologic aspects of this. Because it's counterintuitive – people come to the doctor because they expect a pill, but not everything is solved by a pill. [S02-04-Physicians]<br>• I actually just had a lady that was on insulin for a lot of years and I said, you know, I really just think that this isn't a good idea for you anymore. Let's try stopping your insulin, but let's put you on this. I'm going to come back at these certain intervals, we're going to check your blood sugar, we can adjust these oral medications, but let's do this. And she was completely onboard with it. So I think if you present with an alternative and you are going to give them the follow up for it and the support that they need for it… having them feel like you're going to be there to support them through the changes is important too. [S02-05_NP]<br>• What we've heard with the other physician colleagues on the calls, was - knowing it's a problem and having a plan to alleviate the problem are two different things, right? So what actionable component can you add to "this is a high risk med in dementia, you should be using this instead"? You have to have that "use this instead" easily available. [S01-01-Pharmacist]
	Ongoing dialogue with empathy	• Pushback might be a little strong, but I would say reticence, concern, "I'm not sure this is going to work," and that's where you just kind of say you know what? If this doesn't work, we can

Theme	Subtheme	Illustrative quote
		always add it back, we can always try something else. So just keep a dialogue going and just make sure that they know that you're always available to talk if things don't go well, or if we need to tweak it again. That's where the short term follow up helps, I think… [S02-04-Physician]<br>• Now I do think...with family caregivers you want to have a lot more empathy and you don't want to put too much fear in it [warning about side effects of benzodiazepines]. [S02-06-NP]

Supplement G: Consolidated criteria for reporting qualitative studies (COREQ): 32-item checklist

No. Item	Guide questions/description	Reported on Page #
**Domain 1: Research team and reflexivity**		
Personal Characteristics		
1. Interviewer/facilitator	Which author/s conducted the interview or focus group?	9
2. Credentials	What were the researcher's credentials? E.g. PhD, MD	9
3. Occupation	What was their occupation at the time of the study?	9
4. Gender	Was the researcher male or female?	9
5. Experience and training	What experience or training did the researcher have?	9
Relationship with participants		
6. Relationship established	Was a relationship established prior to study commencement?	8
7. Participant knowledge of the interviewer	What did the participants know about the researcher? e.g. personal goals, reasons for doing the research	8
8. Interviewer characteristics	What characteristics were reported about the interviewer/facilitator? e.g. Bias, assumptions, reasons and interests in the research topic	9
Domain 2: study design		
Theoretical framework		
9. Methodological orientation and Theory	What methodological orientation was stated to underpin the study? e.g. grounded theory, discourse analysis, ethnography, phenomenology, content analysis	9-10
Participant selection		
10. Sampling	How were participants selected? e.g. purposive, convenience, consecutive, snowball	7-8
11. Method of approach	How were participants approached? e.g. face-to-face, telephone, mail, email	8-9
12. Sample size	How many participants were in the study?	8
13. Non-participation	How many people refused to participate or dropped out? Reasons?	NA
Setting		
14. Setting of data collection	Where was the data collected? e.g. home, clinic, workplace	8
15. Presence of non-participants	Was anyone else present besides the participants and researchers?	NA
16. Description of sample	What are the important characteristics of the sample? e.g. demographic data, date	10
Data collection		

17. Interview guide	Were questions, prompts, guides provided by the authors? Was it pilot tested?	8-9
18. Repeat interviews	Were repeat interviews carried out? If yes, how many?	NA
19. Audio/visual recording	Did the research use audio or visual recording to collect the data?	8-9
20. Field notes	Were field notes made during and/or after the inter view or focus group?	NA
21. Duration	What was the duration of the inter views or focus group?	8
22. Data saturation	Was data saturation discussed?	10
23. Transcripts returned	Were transcripts returned to participants for comment and/or correction?	NA
Domain 3: analysis and findings		
Data analysis		
24. Number of data coders	How many data coders coded the data?	7-8
25. Description of the coding tree	Did authors provide a description of the coding tree?	NA
26. Derivation of themes	Were themes identified in advance or derived from the data?	8-9
27. Software	What software, if applicable, was used to manage the data?	8-9
28. Participant checking	Did participants provide feedback on the findings?	NA
Reporting		
29. Quotations presented	Were participant quotations presented to illustrate the themes/findings? Was each quotation identified? e.g. participant number	11-19
30. Data and findings consistent	Was there consistency between the data presented and the findings?	Yes
31. Clarity of major themes	Were major themes clearly presented in the findings?	Yes, 11-19
32. Clarity of minor themes	Is there a description of diverse cases or discussion of minor themes?	Yes, in Supplement F
